# Deleterious phenotypes in wild *Arabidopsis arenosa* populations are common and linked to runs of homozygosity

**DOI:** 10.1093/g3journal/jkad290

**Published:** 2023-12-20

**Authors:** A Cristina Barragan, Maximilian Collenberg, Rebecca Schwab, Sonja Kersten, Merijn H L Kerstens, Doubravka Požárová, Ilja Bezrukov, Felix Bemm, Filip Kolár, Detlef Weigel

**Affiliations:** Department of Molecular Biology, Max Planck Institute for Biology, 72076 Tübingen, Germany; The Sainsbury Laboratory, Norwich NR4 7UH, UK; Department of Molecular Biology, Max Planck Institute for Biology, 72076 Tübingen, Germany; Catalent, 73614 Schorndorf, Germany; Department of Molecular Biology, Max Planck Institute for Biology, 72076 Tübingen, Germany; Department of Molecular Biology, Max Planck Institute for Biology, 72076 Tübingen, Germany; Institute of Plant Breeding, University of Hohenheim, 70599 Stuttgart, Germany; Department of Molecular Biology, Max Planck Institute for Biology, 72076 Tübingen, Germany; Department of Plant Developmental Biology, Wageningen University and Research, 6708 PB, Wageningen, Netherlands; Department of Botany, Faculty of Science, Charles University, 128 01 Prague, Czech Republic; The MAMA AI, 100 00 Prague, Czech Republic; Department of Molecular Biology, Max Planck Institute for Biology, 72076 Tübingen, Germany; Department of Molecular Biology, Max Planck Institute for Biology, 72076 Tübingen, Germany; KWS Saat, 37574 Einbeck, Germany; Department of Botany, Faculty of Science, Charles University, 128 01 Prague, Czech Republic; Department of Molecular Biology, Max Planck Institute for Biology, 72076 Tübingen, Germany

**Keywords:** *Arabidopsis arenosa*, reference genome, wild populations, abnormal phenotypes, runs of homozygosity

## Abstract

In this study, we aimed to systematically assess the frequency at which potentially deleterious phenotypes appear in natural populations of the outcrossing model plant *Arabidopsis arenosa*, and to establish their underlying genetics. For this purpose, we collected seeds from wild *A. arenosa* populations and screened over 2,500 plants for unusual phenotypes in the greenhouse. We repeatedly found plants with obvious phenotypic defects, such as small stature and necrotic or chlorotic leaves, among first-generation progeny of wild *A. arenosa* plants. Such abnormal plants were present in about 10% of maternal sibships, with multiple plants with similar phenotypes in each of these sibships, pointing to a genetic basis of the observed defects. A combination of transcriptome profiling, linkage mapping and genome-wide runs of homozygosity patterns using a newly assembled reference genome indicated a range of underlying genetic architectures associated with phenotypic abnormalities. This included evidence for homozygosity of certain genomic regions, consistent with alleles that are identical by descent being responsible for these defects. Our observations suggest that deleterious alleles with different genetic architectures are segregating at appreciable frequencies in wild *A. arenosa* populations.

## Teaser

Barragan *et al*. survey the progeny of wild *Arabidopsis arenosa* plants to determine the frequency of potentially deleterious phenotypes in natural populations, investigating the genetic basis of several such cases. They find a number of defects likely to decrease fitness in the wild; in some cases, those defects were associated with extended homozygosity at particular genomic regions. This study provides a methodological roadmap for detecting and comparing genetic architectures linked to deleterious phenotypes in wild self-incompatible plant populations.

## Introduction

### Influences on genetic variation and the role of deleterious mutations in outcrossing populations

Standing genetic variation in populations is typically determined by a combination of migration, and the input of new mutations and their removal or retention due to neutral demographic processes or natural selection (Barrett and Schluter 2008). However, there are limits to the effectiveness of natural selection when population sizes are moderate, impeding the removal of detrimental alleles. Practically all wild populations harbor some deleterious genetic variants, which can be due to a number of factors that prevent their timely elimination, including interference with other loci by linkage (Hill and Robertson 1966), balancing selection (Charlesworth and Charlesworth 1999), or epistasis (Perlman 2005). Many of the most severe deleterious mutations are likely to be largely recessive, and they are therefore expected to be most prevalent in relatively small outcrossing populations (Crow and Kimura 1970; Wright *et al*. 2008; Lynch 2010). In outcrossing diploids, such mutations will only be exposed to strong selection when two deleterious alleles at the same locus are combined (Bosse *et al*. 2019). This occurs most easily when alleles that are identical by descent are combined through inbreeding, which can decrease fitness when compared to their noninbred siblings, leading to inbreeding depression (Charlesworth and Willis 2009; Brown and Kelly 2020). In outcrossing species, inbreeding depression is almost certainly more common than in selfing species, and it is often due to a combination of mildly deleterious mutations, which are typically harder to purge (Barrett and Charlesworth 1991; Husband and Schemske 1996; Winn *et al*. 2011), and to recessive large-effect lethal mutations (Charlesworth and Charlesworth 1987; Willis 1992; Lande *et al*. 1994; Remington and O’Malley 2000). Inbreeding depression is therefore considered to be one of the main factors preventing the evolution of self-fertilization (Fisher 1949; Lloyd and Schoen 1992).

## Determining the prevalence and underlying genetics of phenotypic *A. arenosa* Abnormalities

*Arabidopsis arenosa*, which has both diploid and autotetraploid natural populations, is an outcrossing relative of the diploid, selfing *A. thaliana* (Al-Shehbaz and O’Kane 2002), which makes *A. arenosa* an ideal subject for evolutionary studies (Yant and Bomblies 2017; Koch 2019; Gieroń *et al*. 2021). The motivation for this work was to systematically investigate the frequency of aberrant phenotypes among progeny of wild *A. arenosa* plants. Since this species is obligately outcrossing, seeds are the products of natural crosses between different individuals, allowing not only for the detection of deleterious effects that result from homozygosity but also of detrimental epistatic interactions between heterozygous alleles (Johnson 2010). In addition to describing the prevalence of phenotypic abnormalities, we provide a methodological route forward in establishing the potential underlying genetics of deleterious phenotypes using a series of genomic approaches. To this end, we investigated eight diploid populations from the Western Carpathian Mountains, which is a center of genetic diversity of the species (Schmickl *et al*. 2012), and where most genetically variable diploid populations belong to a single lineage (Kolár *et al*. 2016). In addition, we chose diploid *A. arenosa* lineages since these were found to have higher amounts of inbreeding depression than their young polyploid counterparts (Clo and Kolár 2022).

We found a range of apparently deleterious defects in plants grown from seeds collected in the wild and raised in a common environment in the greenhouse. Heritable defects were repeatedly observed and present in the progeny of about one in ten plants. We focused our genetic analyses on four families that produced different phenotypic abnormalities. In two families, we could not identify genomic region(s) that were clearly associated with the observed defects, leaving it unclear as to what the underlying genetics is. In another case, which was associated with different defects, multiple runs of homozygosity (ROH) throughout the genome were shared among abnormal plants. We speculate that various combinations of these regions result in independent abnormalities. Finally, there was one genetically simple case in which a single homozygous genomic region was strongly linked to a distinct deleterious phenotype. Our results suggest that different genomic landscapes, including the presence of ROH, underlie phenotypic defects in the progeny of wild *A. arenosa* plants. In addition, we provide the *A. arenosa* community with a high-quality chromosome-level assembly of this species. Detecting and comparing genetic architectures associated with harmful traits in wild self-incompatible plant populations will contribute to maximizing the efficient utilization of wild genetic material in plant breeding endeavors.

## Material and methods

### Plant sampling, screening, and growth conditions

Over 1,700 *Arabidopsis arenosa* seeds and plant material were sampled from eight different populations in central Slovakia during the summer months under permit number 062-219/18 (Supplementary Table 1). These plants, which we termed F_0_, were phenotypically very variable, ranging from being a few centimeters tall and having few siliques, to plants being half a meter tall and having thousands of siliques. For restriction site-associated DNA sequencing (RAD-seq) of these F_0_ plants, we chose samples covering as much diversity as possible, both phenotypically and geographically (from different patches inside a population) (Supplementary Table 2).

F_1_ plants originating from the seeds collected in the wild were sown in the lab (see growth conditions below). We screened seeds originating from 461 F_0_ plants, and sowed 5–7 F_1_ plants per F_0_, resulting in over 2,500 plants that could be visually screened for gross abnormalities. For families with abnormal phenotypes, these individually were documented, with the most common phenotypes being chlorosis, necrosis, and impaired growth (Supplementary Table 9). If we detected an apparently deleterious phenotype, another batch of 5–7 seeds was sown to confirm the consistency of the phenotype. If the phenotype could be confirmed, then sib-crosses were made between these F_1_ individuals. Wherever possible, we prioritized F_1_ individuals displaying the deleterious phenotype for sibling crosses, even if in a milder form. If all abnormal plants were sterile, healthy-looking siblings were crossed. For linkage mapping, we focused on four F_2_ families, the progeny of F_1_ sib-crosses, with highly consistent phenotypes. Since large numbers of plants were needed, we sequenced F_2_ plants resulting from different parental F_1_ combinations in each family.

Plants were stratified in the dark at 4°C for 5–8 days before planting on soil. Plants were grown on long days (16 h of light) at 16°C or 23°C and at 65% relative humidity under 110–140 μmol m^−2^ s^−1^ light provided by Philips GreenPower TLED modules (Philips Lighting GmbH, Hamburg, Germany) for 6–8 weeks before being moved to greenhouse conditions. The diploid state of each wild *A. arenosa* population sampled was estimated using nQuire (Weiß *et al*. 2018) and confirmed via flow-cytometry (one representative plant per population).

### De novo genome assembly and annotation

A single *A. arenosa* plant from the Strecno population was grown as described above. Fresh green tissue was repeatedly harvested from this individual over several weeks. High-molecular-weight DNA was extracted from ∼50 ml finely ground tissue powder: nuclei were enriched by gentle resuspension of tissue powder in 500 ml fresh and ice-cold isolation buffer (10 mM Tris pH8, 0.1 M KCl, 10 mM ethylenediaminetetraacetic acid, 0.5 M sucrose, 4 mM spermidine, and 1 mM spermine), followed by filtration through two layers of Miracloth, gentle addition of 25 ml isolation buffer containing 20% Triton-X-100, incubation on ice for 15 min, and centrifugation at 3,000 g. Pelleted nuclei were washed with isolation buffer containing 1% Triton-X-100, gently resuspended in 30 ml G2 lysis buffer (Qiagen), and incubated with 50 µg/ml RNaseA (Qiagen) at 37°C for 30 min, followed by proteinase K treatment (200 µg/ml; Qiagen) at 50°C overnight. After centrifugation at 8,000 g, DNA-containing supernatant was purified with Qiagen genomic tip 100 following manufacturer’s instructions. Isopropanol corresponding to 0.7 volumes of the flow-through was gently added, and DNA was pooled with slow tube rotations, then resuspended in elution buffer (EB) buffer (Qiagen) at 4°C. A 20–30 kb XL insert library was sequenced on a PacBio RS II instrument (Pacific Biosciences, Menlo Park, CA, USA). A second >20 kb library (from the same genomic DNA) was sequenced on a PacBio Sequel instrument with Binding Kit 3.0. In addition, a PCR-free library for short-read sequencing was prepared with the NxSeq AmpFREE Low DNA Library Kit (Lucigen, Middleton, WI, USA) according to manufacturer’s instructions, and sequenced on a HiSeq3000 instrument (Illumina, San Diego, USA) in paired-end mode (2 × 150 bp). PacBio long-reads were assembled with Falcon (v0.3.0) (Chin *et al*. 2016). The resulting contigs were polished using the PCR-free reads with Quiver/Arrow using the GenomicConsensus Package (v2.3.2) from PacificBiosciences (https://github.com/PacificBiosciences/) https://github.com/PacificBiosciences/GenomicConsensus), as well as with Pilon (v1.16) (Walker *et al*. 2014). Duplicate contigs were removed using HaploMerger2 (v3.4) (Huang *et al*. 2017). The polished contigs were scaffolded based on the *A. lyrata* assembly (v1) (Hu *et al*. 2011) using REVEAL (v0.2.1) (Linthorst *et al*. 2015). *Arabidopsis arenosa* RNA-seq data (see below) were mapped against the scaffolded genome assembly using hierarchical indexing for spliced alignment of transcripts (v2.0.5) (Kim *et al*. 2015). Subsequently, the mapping results were used as extrinsic RNA evidence when annotating the genome using AUGUSTUS (v3.3.3) (Stanke *et al*. 2006). Transposable elements and repetitive regions were identified using RepeatModeler2 (v2.01) (Flynn *et al*. 2020) and subsequently masked using RepeatMasker (v4.4.0; http://repeatmasker.org). Orthologous genes shared between *A. arenosa* and the *A. thaliana* reference annotation (Araport11) were identified using Orthofinder (v2.4.0) (Emms and Kelly 2019). *Arabidopsis arenosa* and *A. thaliana* protein fasta files were subset to only retain the primary transcript for orthologous assignment using the another gff analysis toolkit (v0.4.0) (https://github.com/NBISweden/AGAT). The final chromosome-level *A. arenosa* reference genome had a total size of 150.0Mb, and a contig N50 of 3.7Mb (Supplementary Table 3). Contig-level statistics were computed with quality assessment tool (v.5.2.0) (Gurevich *et al*. 2013). The Benchmarking Universal Single-Copy Orthologs (BUSCO) (Simão *et al*. 2015) dataset used was embryophyta_odb10. Here, we obtained a BUSCO completeness score of 99% (Supplementary Table 3), reflecting its high-quality both in terms of contiguity and completeness.

### RNA sequencing

**Fig. 1.**
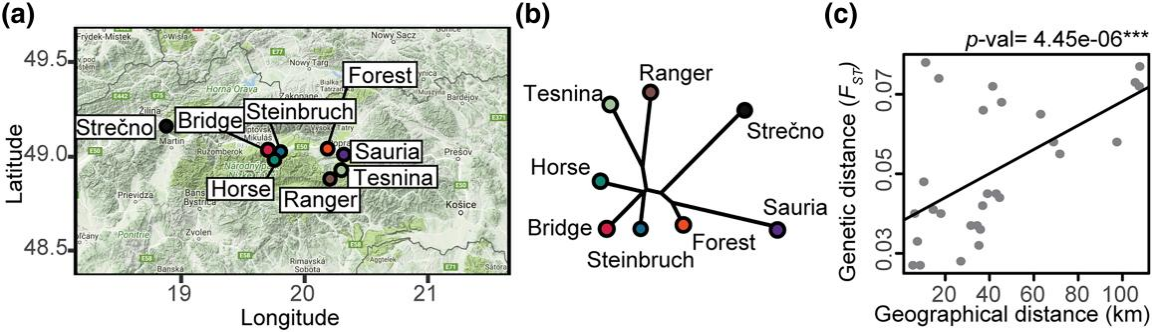
Wild *A. arenosa* populations in the Western Carpathian Mountains are genetically similar and display isolation by distance. a) Locations of the eight studied populations. b) *F_ST_*-based NJ tree. c) Pearson product-moment correlation between genetic (*F_ST_*) and geographical distance among populations.

Five biological replicates of 21-day-old shoots (17 days after germination) of normal and abnormal B772 and A279 plants were collected. At this point, the abnormal phenotypes were clearly visible in both families, but plants expressing the more severe phenotype in the A279 family had not yet started to wither (Fig. 1a). In the A279 family, severely abnormal plants were chosen for expression analysis, while in the B772 family the severity among abnormal plants was not yet evident at the time point of sampling. After RNA extraction (Yaffe *et al*. 2012), sequencing libraries were prepared using the TruSeq Total RNA Kit (Illumina) and the Ribo-Zero Plant Kit (Illumina). Libraries were paired-end sequenced on an Illumina HiSeq3000 instrument (2 × 150 bp). Reads were mapped against our *A. arenosa* reference genome using bowtie2 (v2.2.6) (Langmead and Salzberg 2012). Default parameters were chosen unless mentioned otherwise. Transcript abundance was calculated with RNA-seq by expectation-maximization (v1.2.31) (Li and Dewey 2011). Differential gene expression analyses were performed using DESeq2 (v1.18.1) (Love *et al*. 2014). Genes with less than ten counts over all samples were removed from downstream analyses. Significant changes in gene expression between two genotypes were determined by filtering for genes with a |log_2_FoldChange| > 1 and p_adj_ value < 0.05. Plots were generated using the R package ggplot2 (v3.2.0) (Wickham 2009). Gene Ontology (GO) analyses were performed using AgriGO (Tian *et al*. 2017) with the singular enrichment analysis (SEA) method using default settings, these include a GO significance threshold of *P*-value = 0.05, which has been corrected for multiple-testing using the Yekutieli (false discovery rate, FDR) multiple-testing adjustment method. TAIR9 was used as a gene model while taking as input the *A. thaliana* TAIR10 orthologs of the annotated *A. arenosa* genes. GO results were visualized with reduce + visualize gene ontology (REVIGO) treemap (Supek *et al*. 2011). For clearer visualization, only 15 GO categories with the lowest *P*-values were plotted. Transcriptome-wide expression changes between *A. thaliana* F_1_ plants displaying hybrid necrosis and their parents (Barragan *et al*. 2020) were compared to expression changes between normal and abnormal *A. arenosa* individuals through gene ortholog assignment. For every differentially expressed gene in either the *A. arenosa* or *A. thaliana* dataset, the corresponding orthogroup was extracted. Intersections were then created between these orthogroups containing differentially expressed genes (DEGs) (Supplementary Fig. 2c and Table 4). Lastly, a Monte Carlo simulation was used to predict the distribution of the probability of obtaining a particular number of DEGs between the A279 and B772 families by chance.

### Genotyping-by-sequencing and quantitative trait locus mapping

**Fig. 2.**
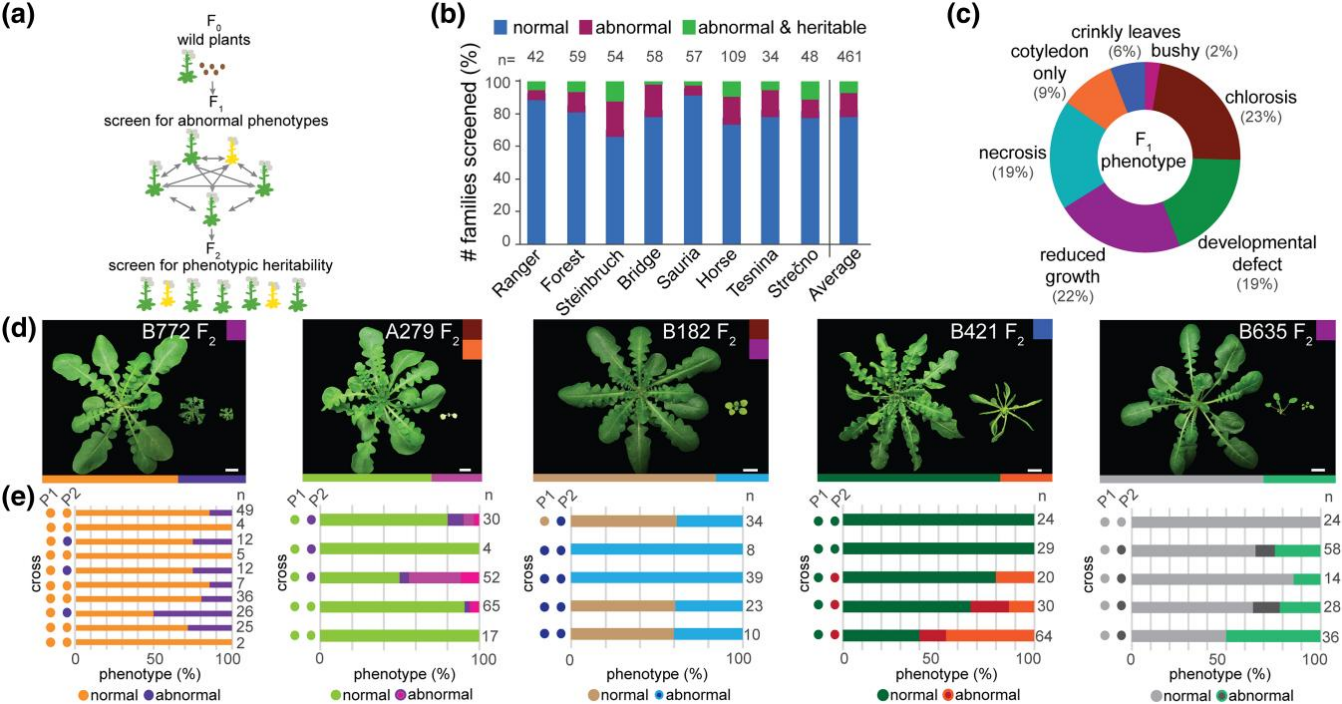
Abnormal *A. arenosa* phenotypes have a genetic basis and are relatively common. a) Experimental design involving the creation of F_2_ populations. F_1_ seeds were collected from wild *A. arenosa* mother plants (F_0_) and were screened for abnormal phenotypes in the lab (yellow plants). If abnormal plants were present, they were crossed with siblings to test whether their phenotype was recapitulated in the following generation (F_2_). b) Percentages of families with abnormal phenotypes that were only observed in the F_1_ generation (“abnormal”), and with phenotypes recapitulated in the F_2_ generation (“abnormal heritable”) per geographic population (see Fig. 2a). The number of F_1_ families screened per population is indicated at the top (n). c) Pie chart showing the most common deleterious phenotypes in the F_1_ plants. Some plants fall under more than 1 category. d) Examples of normal (right) and abnormal, heritable (left) phenotypes from 5 independent families. Phenotype categories are indicated with colors as in C. Plants were 7-weeks-old. Scale bars represent 1 cm. e) Phenotypic distribution of F_2_ plants per family. The parental (P1 and P2) phenotypes are indicated as circles on the left side of each plot and correspond to colors in d, with darker colors for abnormal phenotypes indicating a similar but milder abnormal phenotype (often enabling crossing). The number of F_2_ plants screened per cross is indicated on top (n).

**Fig. 3.**
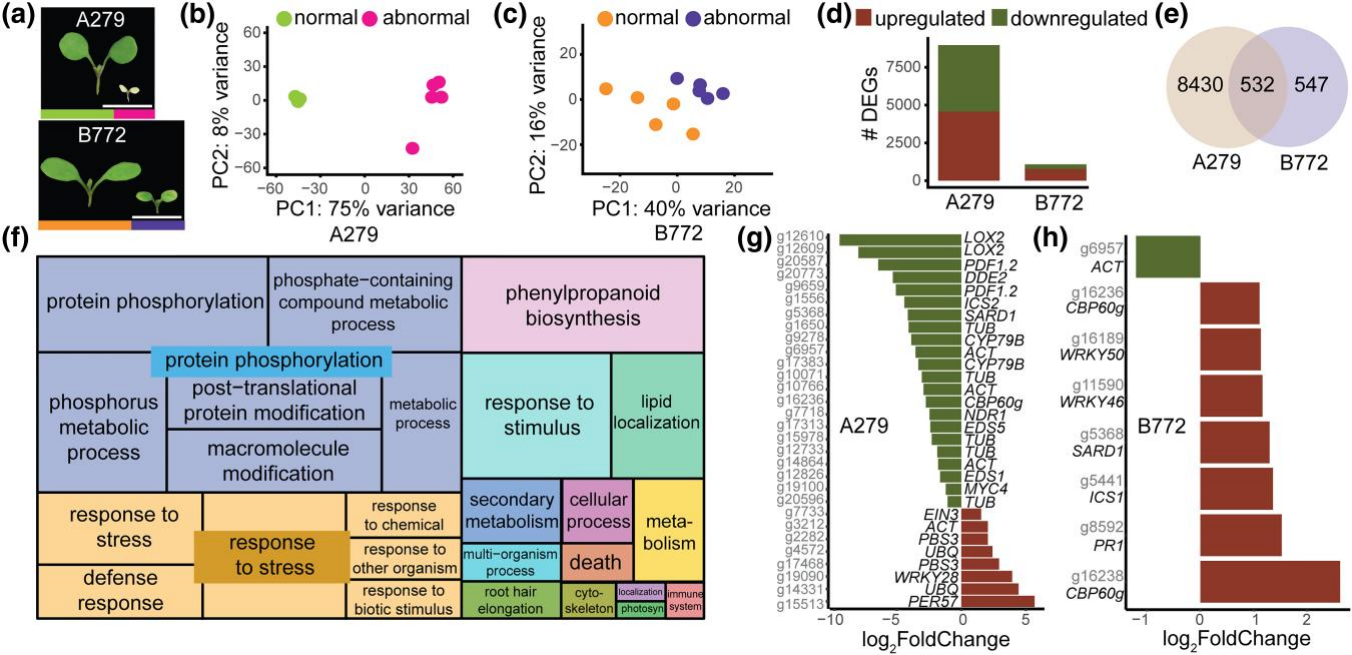
The transcriptional profile of abnormal *A. arenosa* plants is distinct from autoimmune responses. a) At 17 days after germination, normal (green and yellow bars) and abnormal (pink and purple bars) plants from the A279 and B772 families phenotypically differ from each other. Plants were grown at 16°C. Scale bar represents 0.5 cm. b, c) PCA of expression values of all genes after RNA-seq analysis of the A279 and B772 families. The main variance is between normal and abnormal plants. Each dot indicates one biological replicate, with five per family. d) Number of DEGs that are either up- or downregulated in abnormal plants. e) Intersection of DEGs between the A279 and B772 families. f) REVIGO GO treemap of the DEGs in the intersection between the two families. Size of the square represents –log_10_(*P-*value) of each GO term. g, h) –log_2_FoldChange of significant DEGs (|log_2_FoldChange| > 1, p_adj_ value < 0.05). Identifiers (IDs) of *A. arenosa* genes are shown in gray and the names of *A. thaliana* orthologs in black. In case of one-to-many ortholog gene associations, a representative ortholog or a broader term (e.g. *ACT* for all actin orthologs) is shown (Supplementary Table 13).

Genomic DNA was extracted from plants with CTAB (cetyl trimethyl ammonium bromide) buffer (Doyle and Doyle 1987) and then purified through chloroform extraction and isopropanol precipitation (Ashktorab and Cohen 1992). Genotyping-by-sequencing (GBS) of F_0_ individuals by RAD-seq was performed using KpnI tags (Rowan *et al*. 2017). Briefly, libraries were single-end sequenced (1 × 150 bp) on an Illumina HiSeq3000 instrument. Reads were processed with Stacks (v1.35) (Catchen *et al*. 2013) and mapped to our *A. arenosa* reference (Supplementary Table 3) with bwa-mem (v0.7.15) (Li 2013), variant calling was performed with genome analysis toolkit (GATK) (v4.0) (McKenna *et al*. 2010). SNP filtering was performed with VCFtools (v0.1.14) (Danecek *et al*. 2011). Filtering criteria for F_0_ individuals were bi-allelic SNPs only, SNPs with at most 10% missing data, individuals with less than 40% missing data, and SNPs with a minimum depth of 4 and a maximum depth of 80. SNPs were then linkage disequilibrium (LD)-pruned (−indep-pairwise 50 5 0.5) with PLINK (v1.90) (Purcell *et al*. 2007). Downstream analyses were based on 345 individuals and 5,199 markers with an average depth per position of 45 (Fig. 2 and Supplementary Fig. 1). For F_2_ individuals, which were generated by intercrossing 5–7 F_1_ individuals with each other, SNPs with 30% missing data, individuals with less than 40% missing data, and SNPs with a minimum allele frequency of 0.01 were removed, and so were those with a depth of more than 100. F_2_ plants were used as mapping populations for quantitative trait locus (QTL) analyses, which were performed with R/qtl (Broman *et al*. 2003). Here, the genome was scanned with a single QTL mode (scanone) using the expectation–maximization (EM) algorithm and the “kosambi” function for estimating the genetic map. QTL analyses were based on 227 individuals and 11,858 markers with an average depth per position of 29 (B772 family), 162 individuals, and 9,064 markers with an average depth per position of 23 (B182 family), 183 individuals, and 14,672 markers with an average depth per position of 25 (A279 family) and 271 individuals and 6,110 markers with an average depth per position of 25 (B635 family) (Fig. 3 and Supplementary Fig. 3).

### Whole-genome sequencing

Libraries from 52 F_2_ individuals (23 normal and 29 abnormal) from the B772 family, 28 F_2_ individuals (7 abnormal and 21 normal) from the A279 family, 37 F_2_ individuals (17 normal and 20 abnormal) from the B182 family, and 40 wild F_0_ individuals from the Strecno population were prepared (Picelli *et al*. 2014) and paired-end sequenced on an Illumina HiSeq3000 instrument. Reads were processed with Stacks (v1.35) (Catchen *et al*. 2013) and mapped to our *A. arenosa* reference genome (Supplementary Table 3) with bwa-mem (v0.7.15) (Li 2013), variant calling was performed with GATK (v4) (McKenna *et al*. 2010). SNPs with at most 30% missing data (50% for the A279 family), individuals with less than 35% missing data, SNPs with a minimum allele frequency of 0.01, a minimum depth of 4 and a maximum depth of 100 were filtered out, resulting in 43,885 SNPs (B772 F_2_), 138,620 SNPs (A279 F_2_), 45,550 SNPs (B182 F_2_), and 57,922 SNPs (Strecno F_0_). After LD-pruning (−indep-pairwise 50 5 0.5) 3,737 SNPs with an average depth per position of 16 (B772 F_2_), 7,037 SNPs with an average depth per position of 18 (A279 F_2_), 4,987 SNPs with an average depth per position of 13 (B182 F_2_) and 3,903 SNPs with an average depth per position of 10 (Strecno F_0_) remained.

### Population genetic analyses

**Fig. 4.**
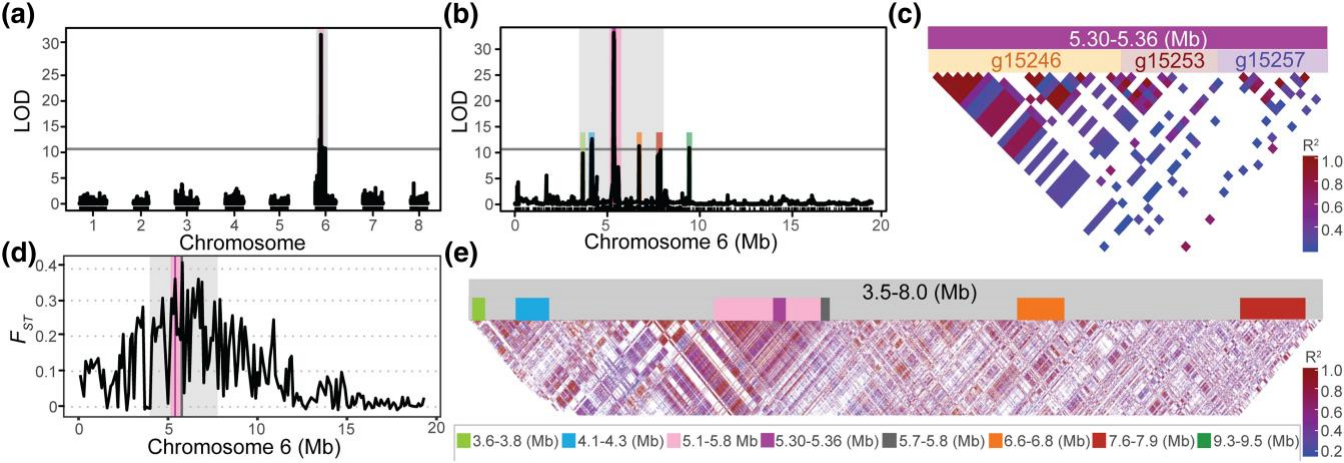
A single genomic region is linked to a deleterious *A. arenosa* phenotype. a, b) QTL analysis of B772 F_2_ plants. A peak is found on chromosome 6 (5.30–5.36 Mb). Horizontal lines indicate 0.05 significance threshold established with 1,000 permutations. The 3.5–8.0 Mb region of chromosome 6 is highlighted in gray. c) LD across the 60 kb B772 QTL region between 5.30–5.36 Mb in chromosome 6. Strong linkage is observed in genes found between g15246 and g15253 and, to a lesser extent, to those found until g15257. The centromere is to the left. d) Fixation index (*F_ST_*) between normal and abnormal B772 F_2_ plants across chromosome six. The 3.5–8.0 Mb region is highlighted in gray, the 5.30–5.36 Mb region in magenta and the 5.1–5.8 region in pink. e) LD plot from the 3.5–8.0 Mb region in chromosome 6 from 52 B772 F_2_ individuals. The region comprising both the highest LOD (magenta) and *F_ST_* (dark gray) is under LD (pink), an indication of reduced recombination. Other colors indicate positions of QTL peaks in B as well as the maximum *F_ST_* value in D.

Principal component analyses were calculated with smartPCA (Patterson *et al*. 2006). *F_ST_* was determined with VCFtools (v0.1.14). Maps were created with the R-packages maps (v3.3) and ggmap (v3.0) (Kahle and Wickham 2013). Effective population sizes were estimated with NeEstimator (v2) (Do *et al*. 2014), using the linkage disequilibrium model. LD (r^2^), inbreeding coefficient (*F*), and ROH were calculated with PLINK (v1.90). For ROH identification, both default parameters (−homozyg-window-het = 1 and –homozyg-window-missing = 5, Fig. 4c and Supplementary Table 5) as well as slightly less stringent filters (−homozyg-window-het = 2 and –homozyg-window-missing = 15, Supplementary Fig. 5, h and l, Table 6 and 7) were used. Sequences were aligned with MUSCLE (v3.8.31) (Edgar 2004) and then visualized with Aliview (v1.18.1) (Larsson 2014). Neighbor-joining (NJ) trees were estimated with either Jalview (Clamp *et al*. 2004) or Fastphylo (v1.0.1) (Khan *et al*. 2013), and visualized in Figtree (v1.4.3) (Rambaut 2012). Genotypes from VCF files were visualized with Genotype Plot (https://github.com/JimWhiting91/genotype_plot). SnpEff (Cingolani *et al*. 2012) was used to predict the effects of variants and identify high-impact mutations using our *A. arenosa* annotation as a reference.

## Results

### Wild *A. arenosa* populations in the western Carpathian mountains are genetically similar and display isolation by distance

Plant material and seeds were collected, nondestructively, from most individuals from each of the 8 different diploid *A. arenosa* populations in central Slovakia (Fig. 1a, Fig. 2a and Supplementary Table 1). The number of sampled plants was therefore proportional to the size of the sampled populations (Supplementary Fig. 1a). We collected at least a dozen, but usually hundreds of seeds per plant, at a time when most seeds had already been shed, ensuring the undisturbed persistence of all populations. Seeds from a single mother plant are either siblings (if they share the same pollen donor) or half sibs (if they do not). We will refer to the immediate as well as all later-generation progeny from a single mother plant as “families’.

We proceeded to examine the genetic relationship between individuals from the studied populations, and individually genotyped 345 of the 1,768 sampled mother plants by RAD-seq (Rowan *et al*. 2017) (Supplementary Table 1). To facilitate population genetic studies, we generated a near chromosome-scale *A. arenosa* reference genome (Supplementary Table 3). The individual sequenced for this purpose came from the Strecno population.

We observed isolation by distance between the eight studied populations (Fig. 2, b and c). A Principal component analysis (PCA) revealed that variance between individuals from different populations is explained by multiple-small-effect components (Supplementary Fig. 1c). Genetic distance (*F_ST_*) between individuals from different populations was slightly lower compared to previous reports on populations of the close relative *A. lyrata* (Gaudeul *et al*. 2007; Hämälä and Savolainen 2019), potentially because our populations are geographically closer to each other than the *A. lyrata* populations (Supplementary Fig. 1d). Nucleotide diversity among individuals from the same *A. arenosa* populations were similar across all pairwise comparisons (Supplementary Fig. 1e). Taken together, individuals from the studied *A. arenosa* populations are genetically relatively similar to each other, with differences increasing with distance, following a typical isolation-by-distance pattern.

### Heritable deleterious phenotypes Among wild *A. arenosa* Plants Are Relatively Common

**Fig. 5.**
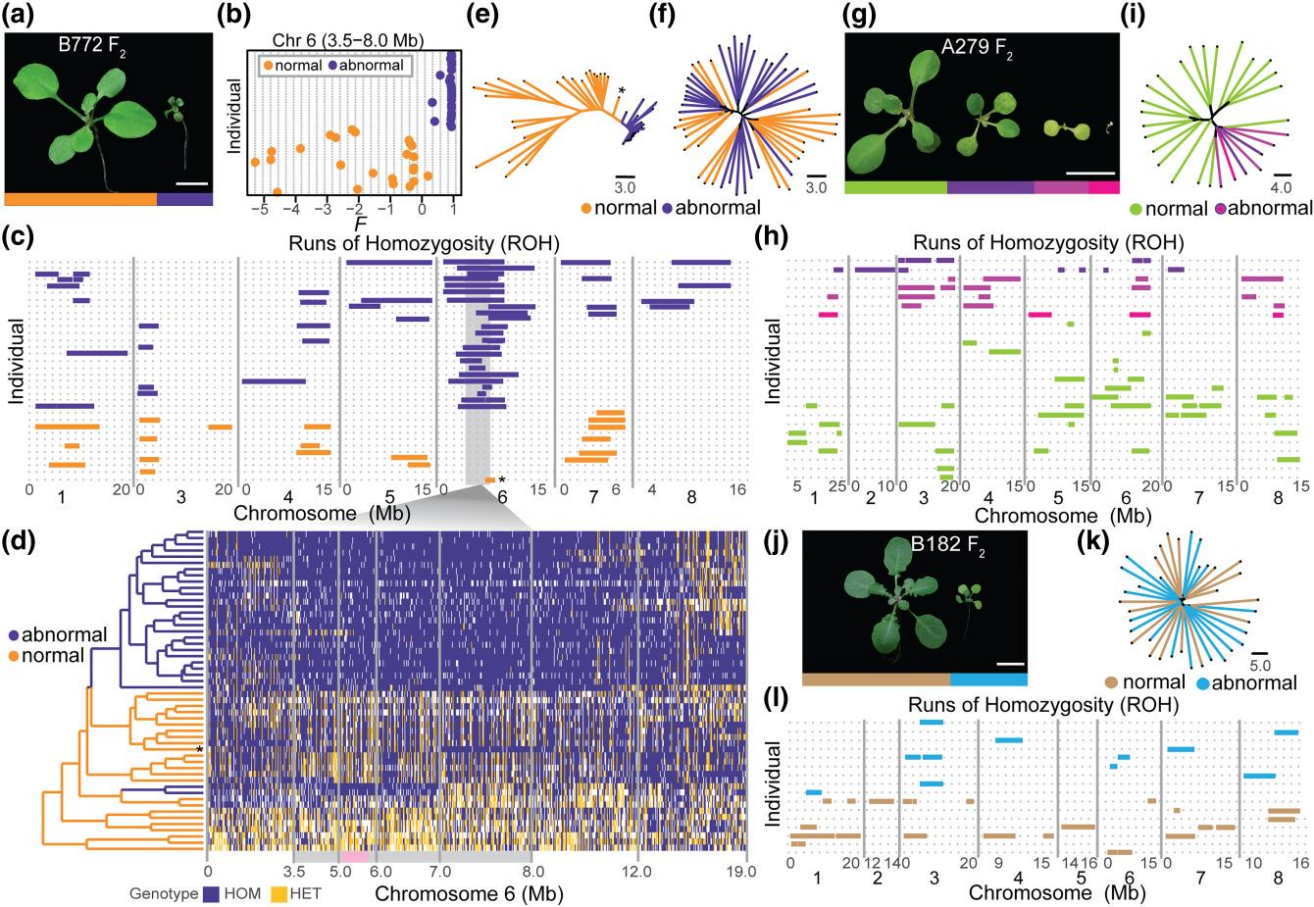
Deleterious phenotypes are linked to increased homozygosity at specific genomic regions. a)-h B772 family. a) Normal (yellow bar) and abnormal (purple bar) appearing plants. b) Inbreeding coefficient (*F*) of normal and abnormal plants for the 3.5–8.0 Mb region on chromosome 6. Abnormal plants have a much higher *F*. c) Genome-wide ROH. Twenty-three plants with an abnormal phenotype share ROH in chromosome 6, while only one normal-appearing plant (indicated by an asterisk) had a short ROH in this region. The 3.5–8.0 Mb region is marked in gray. Only plants with at least one ROH are shown. d) Genotype calls from normal and abnormal plants in chromosome 6 show high homozygosity in abnormal individuals. LD block overlapping the QTL (see Fig. 3) in pink. HOM ALT, markers homozygous for nonreference (alternative) allele in purple; HET, heterozygous genotype calls in yellow. Individuals cluster by chromosome-wide similarity. The colors in the dendrogram indicate the plant phenotype. e) NJ tree of the 3.5–8.0 Mb region on chromosome 6. Individuals cluster by phenotype. Asterisk indicates the same individual as in c. f) NJ tree of sequence differences across the entire genome. Individuals do not cluster by phenotype. Branch lengths as nucleotide substitutions per site are indicated. g–h) A279 family. g) Normal (green bar), mildly affected (purple bar), strongly chlorotic (light purple bar), and severely affected albino (pink bar) plants. h) Genome-wide ROH. No ROH is shared across all abnormal individuals. i) Genome-wide NJ tree. Individuals cluster by phenotype. j–l) B182 family. j) Normal (brown bar) and abnormal (blue bar) looking plants. k) Genome-wide NJ tree. Individuals do not cluster by phenotype. l) Genome-wide ROH. ROH are not linked to the abnormal phenotype. Stringent ROH parameters are shown in d, and less stringent parameters in H and L (see Methods). Plants were between 3- and 5-weeks-old. Scale bars represent 1 cm.

To study the frequency at which deleterious phenotypes segregate among plants resulting from open-pollinated crosses among co-occurring individuals in the wild, we sowed 5 to 7 seeds from 461 families originating from all 8 sampled populations (Supplementary Table 8). We then screened these F_1_ plants for phenotypes that are likely to reduce fitness in the field, such as developmental defects and necrosis (Fig. 5a and Supplementary Table 8). If heritable, these phenotypes could be caused by the presence of deleterious alleles at single loci, by the cumulative action of such deleterious alleles, by deleterious epistatic interactions between alleles of different loci, or by a combination of any of these.

In 86 of the 461 families (18%), at least one of the 5–7 F_1_ plants showed obvious phenotypic abnormalities (Fig. 5b and Supplementary Table 8), with the most common being chlorosis, necrosis, and impaired growth (Fig. 5c and Supplementary Table 9). The severity of defects ranged from very mild and disappearing with age to plants not developing past the cotyledon stage and dying shortly after germination (Fig. 5d). We created F_2_ populations by intercrossing F_1_ siblings for all 86 families in which deleterious phenotypes were observed to test if these phenotypes were heritable (Fig. 5a). For families where all abnormal plants were sterile, crosses among normal-looking or mildly abnormal-looking plants were performed (Fig. 5e and Supplementary Table 10). A total of 37 out of the 86 families tested (43%) produced affected offspring that resembled the abnormal parental individuals, indicating that almost half of the initially observed deleterious phenotypes were heritable and likely to have a genetic basis.

Although the eight *A. arenosa* populations studied are genetically similar to each other, the fraction of families with abnormal heritable phenotypes varied considerably between the sampled populations, from less than 2% in the Bridge population to 7% in the Steinbruch population (Fig. 5b and Supplementary Table 8). We did not find a significant correlation between the frequency of abnormal heritable phenotypes and the effective population size or the inbreeding coefficient of each population (Supplementary Table 8). Taken together, these results show that abnormal, heritable phenotypes appear at appreciable frequencies in progeny of wild *A. arenosa* populations.

### The transcriptional profile of abnormal *A. arenosa* plants is distinct from autoimmune responses

In *A. thaliana*, the most common morphological defects observed in F_1_ progeny after crossing different wild individuals result from autoimmunity, which was first discovered by transcriptional profiling (Bomblies *et al*. 2007). We therefore pursued a similar approach for 2 of ou*r A. arenosa* families, 279 from the Sauria population and B772 from the Strecno population, which consistently included plants with stunted growth and necrotic leaves (Fig. 1a, 4, a and g, 5d). To allow for comparability between families, tissue was harvested from five normal and five abnormal plants from both families 17 days after germination. A PCA showed that most of the variance in gene expression was driven by the difference between plants with and without the abnormal phenotypes (Fig. 1, b and c). This was particularly obvious in the A279 family, where the abnormal phenotype was more pronounced. Accordingly, in abnormal plants of the A279 family, 8,962 genes out of 22,640 annotated genes were differentially expressed, whereas in B772 only 1,079 genes were differentially expressed (Fig. 1d). There was substantial overlap (532 genes) between the DEGs between the A279 and the B772 families (Fig. 1e); this exceeded the median of what was expected by chance by 20%. A GO analysis of these overlapping DEGs revealed these to be enriched for the terms “protein phosphorylation” and “response to stress” (Fig. 1f and Supplementary Table 11). Genes in these two categories were largely upregulated in abnormal plants (Supplementary Table 12). We also assessed the top 100 (Supplementary Fig. 2, a and b) and 500 DEGs for each family separately (Supplementary Table 12). Both sets were enriched for similar terms to those of their intersection, with the A279 DEGs being additionally enriched for “response to abiotic stimulus” and “postembryonic development.”

The stunted and necrotic *A. arenosa* plants shared morphological similarities with *A. thaliana* plants suffering from autoimmunity, characterized by abnormal expression of many immune genes (Bomblies *et al*. 2007; Chae *et al*. 2014; Barragan *et al*. 2020). However, genes that were differentially expressed in autoimmune *A. thaliana* plants, but not in abnormal *A. arenosa* plants were enriched for the GO terms “immune response” and “cell death” (Supplementary Fig. 2c and Table 13). Furthermore, we specifically investigated the expression of *A. arenosa* orthologs of genes in these GO categories, as well as of other marker genes linked to growth, including synthesis and response to phytohormones (Papadopoulou *et al*. 2018) (Supplementary Table 13). For both *A. arenosa* families, few of the 35 selected genes were markedly altered in their expression (Fig. 1g). In addition, genes encoding intracellular nucleotide binding site-leucine-rich repeat immune receptors (NLRs) were much less induced than previously seen in autoimmune *A. thaliana* individuals (Supplementary Fig. 5, d and e and Table 14). Lastly, since autoimmune defects are often suppressed by elevated temperature (van Wersch *et al*. 2016), we compared plants from the A279 and B772 families grown at either 16°C or 23°C to further confirm that the deleterious phenotypes that segregate in our *A. arenosa* families are unlikely to be immunity related. The phenotypes at both temperatures were very similar (Supplementary Fig. 5, f–i). These results indicate that the dwarfism and necrosis found in two *A. arenosa* families are unlikely to result from inappropriate activation of the immune system.

While inbreeding can lead to population-specific transcriptional changes, a common feature in the closely related species *A. lyrata* is the overrepresentation of genes belonging to GO terms associated with stress responses and metabolic activity (Menzel *et al*. 2015). Since the observed deleterious phenotypes in *A. arenosa* might be caused by extended homozygosity brought on by inbreeding, we compared GO terms overrepresented in DEGs between normal and abnormal *A. arenosa* and between inbred and outcrossed *Arabidopsis lyrata*. While these GO categories “stress responses” and “metabolic activity” are quite general, they were overrepresented among DEGs in our *A. arenosa* A279 and B772 families (Supplementary Table 12), potentially hinting that at least some of the observed abnormalities resulting from inbreeding.

### A single genomic region is linked to a deleterious *A. arenosa* phenotype

To identify the genetic basis of the deleterious phenotypes found segregating in *A. arenosa*, both normal and abnormal plants from four independent F_2_ families (B772, A279, B635, and B182) were individually genotyped by RAD-seq (Rowan *et al*. 2017) (Fig. 5a). These families were chosen for further analysis because abnormal phenotypes were particularly consistent in these families and because phenotypes of some affected individuals were sufficiently mild to enable sibling crosses. QTL analysis did not reveal associations between specific genomic regions and the abnormal phenotypes in three out of the four families studied (Supplementary Fig. 3, a–d).

In the B772 family, however, we identified a single high-confidence QTL on chromosome 6. The QTL interval included 12 annotated genes at 5.30–5.36 Mb, with the highest log10 likelihood ratio (LOD) score of 32.9 at 5.34 Mb (Fig. 3, a and b and Supplementary Table 15). Recombination in this interval was limited in the B772 family, especially toward the centromere side of the QTL interval (Fig. 3c). None of these 12 genes was significantly (|log_2_FoldChange| > 1, p_adj_ value < 0.05) differentially expressed between normal and abnormal looking plants (Supplementary Table 15). In a larger genomic region spanning 3.5–8.0 Mb on chromosome 6, 4 additional, marginally significant QTL peaks were found (Fig. 3b). This larger 4.5 Mb region contained 927 genes (Supplementary Table 16), which were enriched for the GO terms “cell wall biogenesis” and “carbohydrate biosynthesis” (Supplementary Table 17). Of the 927 genes, 49 were differentially expressed between normal and abnormal-looking plants (Supplementary Table 18), but these were not enriched for any particular GO term.

To examine the genomic region associated with the deleterious phenotype in the B772 family more closely, we whole-genome-sequenced 52 F_2_ individuals, 24 with a normal and 28 with an abnormal phenotype, and then calculated the fixation index (*F_ST_*) as an indicator of genetic differences between the two phenotypic groups in this family. In accordance with the QTL analysis described above, a peak in the first half chromosome 6 was observed, with a maximal *F_ST_* value of 0.41 between 5.7–5.8 Mb, followed by 5.3–5.4 Mb with an *F_ST_* value of 0.36 (Fig. 3d). These 2 regions are part of the same 700 kb long linkage block (5.1–5.8 Mb) showing reduced recombination (Fig. 3e). No other regions with elevated *F_ST_* values were observed across the entire genome (Supplementary Fig. 4e). Taken together, this indicates that a region in the first half of chromosome 6, especially a particular LD block, is genetically differentiated between phenotypically normal and abnormal plants and potentially harbors the causal locus or loci for the abnormal phenotype.

### Runs of homozygosity are common in wild individuals from the Strečno population

We found a large 700 kb LD block on chromosome 6 to cosegregate with phenotypic abnormalities in the B772 family. The primary cause of these defects may be increased homozygosity in this region, thereby exposing one or multiple deleterious recessive mutations, incompatibility between two alleles of the same gene, or negative epistasis between two closely linked genes.

To distinguish between these possibilities, and the following leads from the RNA-seq analysis, we investigated the inbreeding coefficient (*F*) as a proxy for homozygosity within the two phenotypic groups (normal and abnormal) in the 3.5–8.0 Mb region surrounding the LD block on chromosome 6 (Fig. 4a). Notably, abnormal individuals showed a higher *F* than normal ones (Fig. 4b and Supplementary Table 19). As a control, *F* was calculated for both a similarly sized genomic region on chromosome 1, as well as for the entire genome, but no differences in inbreeding levels between normal and abnormal plants were seen outside the LD block on chromosome 6 (Supplementary Fig. 4, a and b and Table 19).

We also searched for ROH, which would hint at inbreeding depression as a potential cause underlying the deleterious B772 phenotype. ROH was indeed identified throughout chromosome 6 in 82–100% of abnormal plants, depending on the parameter settings (Fig. 4c and Supplementary Table 5). For normal-appearing individuals, only short ROH was identified in this region in 1–3 (4–12.5%) plants, depending on the parameters chosen (Supplementary Table 5). For other regions in the genome, ROH were both less frequent and not associated with the occurrence of the abnormal phenotype (Fig. 4c and Supplementary Table 5). High levels of homozygosity present in abnormal individuals throughout chromosome 6 were confirmed by direct visual inspection of genotype calls (Fig. 4d). An association between homozygosity and the abnormal phenotype was also not observed in any other region of the genome (Supplementary Fig. 4c). As expected, sequences from 3.5 to 8.0 Mb region, shown to be differentiated between normal and abnormal plants, separated normal and abnormal individuals in a NJ tree, which confirms high sequence similarity among abnormal siblings (Fig. 4e), which was not observed for the whole genome (Fig. 4f), or for the control region on chromosome 1 (Supplementary Fig. 4, c and d).

Since recessive alleles appear to be causal, we aimed to identify candidate genes underlying the abnormal phenotype inside the 3.5–8.0 Mb ROH by looking for high-impact mutations. We found seven such mutations in the 3.5–8.0 Mb region of chromosome 6, 6 of which were homozygous, although not exclusively, in abnormal plants (Supplementary Table 20). This indicates that a mutation in more than one gene in this region may be responsible for the phenotypic defects. We can of course not exclude that mutations other than those defined as having high impact are relevant, either solely or in addition to the identified high-impact mutations.

For the families where we did not find a clear QTL associated with phenotypic abnormalities, we wondered whether simultaneous homozygosity in multiple regions might cause the abnormal phenotypes, possibly explaining why no QTLs were identified before. To investigate this, we sequenced the complete genomes of individuals with a range of different defects from the A279 family. Of the 28 sequenced individuals, two were mildly chlorotic, but of normal size, four were strongly chlorotic, dwarfed, and did not develop true leaves, and one was a very small albino (Fig. 4g). We calculated *F* in 5 Mb windows across the whole genome and saw that there were multiple regions where abnormal plants tended to have a higher *F* than normal plants (Supplementary Fig. 4e and Table 21). We also calculated genome-wide *F*, and abnormal plants were part of the upper quartile of values (Supplementary Fig. 4f and Table 21). Different from the B772 family, we found ROH in similar proportions in both normal and abnormal plants (Fig. 4h and Supplementary Table 6). There was no single ROH that was shared between all abnormal A279 individuals, however, which was also apparent from direct visual inspection of genotype calls (Supplementary Fig. 4g). These data are consistent with different combinations of unlinked ROH giving rise to different phenotypic defects, but additional individuals would need to be analyzed to support this assertion more fully. Unlike B772, abnormal plants clustered together when whole-genome sequence variation was considered (Fig. 4i), further hinting at sequences throughout the genome contributing to the phenotypic abnormalities.

Finally, we whole-genome sequenced 17 normal and 20 abnormal individuals from a third family, B182 from the Horse population, which consistently segregated plants with a uniform, chlorotic dwarf phenotype (Fig. 4j), for which we also had not detected a clear QTL. Unlike the two previous families, no relationship between the abnormal phenotype and overall inbreeding levels, or ROH, was found, nor were the abnormal plants genetically more similar to each other than to normal individuals (Fig. 4, j–l, Supplementary Fig. 4, h and i, Table 7 and 22). This makes us suspect a more complex genetic architecture underlying the phenotypic abnormalities in the B182 family, perhaps even involving nuclear-cytoplasmic interactions. Such a scenario would be in agreement with many more than a quarter of plants, as expected for a single causal genomic region, having an abnormal phenotype. To summarize, we found high homozygosity present in particular genomic regions to be associated with deleterious phenotypes segregating in two wild *A. arenosa* families but could not link any regions of the nuclear genome to phenotypic abnormalities in the third family studied in detail.

### Homozygosity in the region underlying phenotypic abnormalities is rare in the wild

**Fig. 6.**
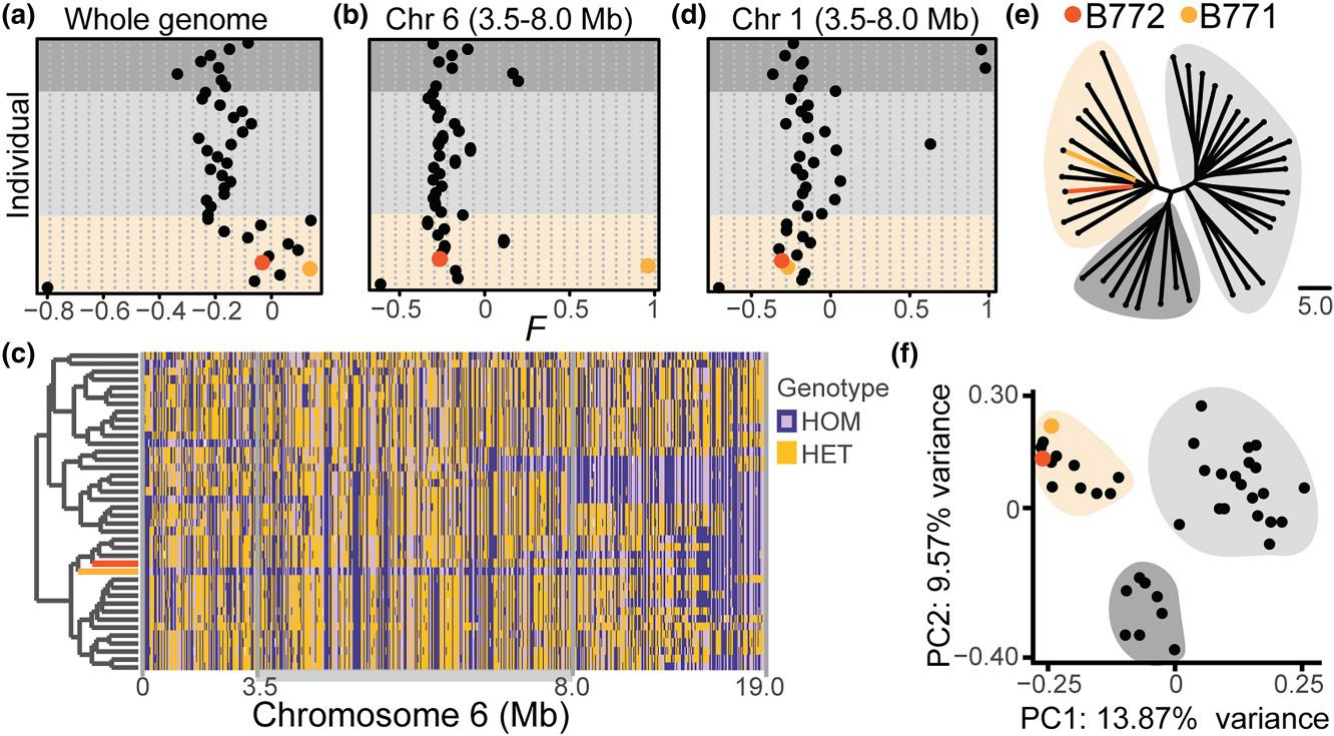
ROH are common in wild *A. arenosa* individuals from the Strecno population. a, b, and d) Inbreeding coefficient (*F*) of wild plants collected from Strecno for the whole genome (a), the 3.5–8.0 Mb region of chromosome 6 (b), and the 3.5–8.0 Mb region of chromosome 1, a control region unlinked to abnormal phenotypes in the greenhouse (d). Individuals follow the same order in the three plots. The B771 F_0_ plant (dark yellow), but not the B772 F_0_ (orange) had a high *F* value in the 3.5–8.0 Mb region of chromosome 6. Background colors as in e. c) Genome-wide genotype calls from the 40 sequenced wild Strecno plants. HOM ALT, markers homozygous for nonreference (alternative) allele; HOM REF, markers homozygous for reference allele; HET, heterozygous genotype calls. Individuals clustered by chromosome-wide similarity. The B771 and B772 F_0_ plants (indicated by colored lines on the far left) have similar genotype calls, with B771 F_0_ but not B772 F_0_ being highly homozygous throughout chromosome 6. The 3.5–8.0 Mb region is highlighted in gray. e) Genome-wide NJ tree. Three genetic groups are visible (dark gray, light gray, and yellow), with B771 and B772 F_0_ plants belonging to the same group (Supplementary Table 23). Branch lengths in nucleotide substitutions are indicated. f) Genome-wide PCA of the 40 sequenced individuals, each represented by a dot. The same individual clustering is observed as in e.

To assess how often ROH was observed across chromosome 6 in wild individuals from the Strecno population, where the original B772 mother plant was collected from, especially in the 3.5–8.0 Mb region associated with the presence of the abnormal phenotype, we whole-genome sequenced 40 individuals. Genome-wide *F* was not unusually high in the original B772 F_0_ individual collected from the wild, compared to other Strecno individuals (Fig. 6aand Supplementary Table 23). The 3.5–8.0 Mb region of chromosome 6, which we found to be homozygous in abnormal B772 F_2_ plants, was not exceptionally homozygous in the B772 F_0_ plant either (Fig. 6b). This agrees with our observation of the B772 F_0_ plant appearing normal in the field. Four individuals showed positive *F* values in this region. This was not due to missing data, which was below 0.02% across chromosome 6 for all individuals (Supplementary Table 23). The most homozygous individual for this region was the B771 F_0_ plant, which had been found right next to the B772 individual, with an *F* value of almost 1 (Fig. 6b and Supplementary Table 23). Extensive homozygosity in the B771 F_0_ individual across chromosome 6 was confirmed by directly inspecting genotype calls (Fig. 6c). As a control, *F* was calculated for the 3.5–8.0 Mb region in chromosome 1 across all 40 plants (Fig. 6d). Neither B772 nor B771 F_0_ individuals had high *F* in this region, but three other plants had, indicating that homozygous stretches across the genome are relatively common in wild individuals from this population. This was again confirmed by directly inspecting genotype calls (Supplementary Fig. 5a). A genome-wide NJ tree, as well as a PCA, placed B771 and B772 F_0_ plants near each other. This also revealed three distinct genetic groups present in the Strecno population (Fig. 6, e and f, Supplementary Table 23). In brief, we found extended homozygosity to be common in wild *A. arenosa* plants. In the case of the B772 F_0_ plant, however, the 3.5–8.0 Mb region of chromosome 6 associated with the abnormal phenotype was not highly homozygous. A possible explanation is that fertilization by closely related pollen donors (such as B771 F_0_, collected next to B772 F_0_) resulted in the high homozygosity that we observed in later B772 generations raised in the greenhouse.

## Discussion

### Insufficient genetic diversity may result in inbreeding depression in wild *A. arenosa* populations

Excessive genetic divergence between individuals at particular loci can lead to allelic mismatches in their progeny. Such deleterious epistatic genetic interactions are often a consequence of breaking up co-evolved gene complexes (Lynch 1991). This is what has been observed in the *A. arenosa* congener *A. thaliana*, where a mismatch between divergent alleles of immune-related loci results in hybrid necrosis (Li and Weigel 2021). This genetic incompatibility syndrome is by far the most common F_1_ hybrid weakness phenotype observed in this species under laboratory conditions (Bomblies *et al*. 2007).

In contrast to high divergence at particular loci being deleterious, high genetic similarity between outbred individuals increases the likelihood of their progeny inheriting identical alleles from both parents, thereby exposing unpurged recessive deleterious mutations and potentially resulting in inbreeding depression (Charlesworth and Charlesworth 1999; Charlesworth and Willis 2009). Inbreeding depression is relatively common in outcrossing plant species (Moyle *et al*. 2014), and at least some of the deleterious *A. arenosa* phenotypes we observed may be attributed to inbreeding depression because they are linked to extended homozygosity. In *A. arenosa*, these deleterious and heritable phenotypes were common and observed in about one in ten maternal sibships. For comparison, in a study of another wild, outcrossing Brassicaceae species, *Leavenworthia alabamica*, a third of all families were lost after one generation of selfing (Baldwin and Schoen 2019), highlighting the strong impact inbreeding can have on plant fitness. That study reported around 3% of abnormal phenotypes among all individuals after outcrossing. Of 2,640 *A. arenosa* plants raised from seeds collected in the wild, we observed morphological abnormalities in 157 plants (5.9%), which is higher, but not hugely different.

We note, however, that we have focused on progeny that result from crosses between naturally occurring individuals within local populations. Pressure for co-evolution between genomic loci is expected to be higher among co-occurring individuals. Since breaking up co-evolved loci can result in unfit progeny, genetic mismatches between individuals originating from different populations, may be more common. To address this, we performed a limited number of crosses (*n* = 33) in the greenhouse between *A. arenosa* individuals from different populations. Here, abnormal phenotypes were common as well, with half of all crosses segregating abnormal phenotypes (Supplementary Table 24). Further study will reveal whether these phenotypes are linked to allelic mismatches with a simple genetic architecture, as is common for hybrid necrosis (Li and Weigel 2021).

### Different genetic architectures underlie phenotypic abnormalities in *A. arenosa*

Of the *A. arenosa* families we investigated more in detail, relatively simple genetics underlying phenotypic abnormalities were only found in the case of the B772 family. Here, a single clear QTL was observed, which was part of a larger LD block, suggesting reduced recombination in this genomic region, a feature that has been associated with increased accumulation of deleterious mutations in other species (Haddrill *et al*. 2007; Rieseberg and Willis 2007; Renaut and Rieseberg 2015; Rodgers-Melnick *et al*. 2015; Zhang *et al*. 2019). This specific QTL region was found to be homozygous in all abnormal plants, but largely heterozygous in all normal plants. Whether the phenotypic abnormalities in the B772 family are due to a single large-effect deleterious mutation or multiple mutations in genes in the QTL interval is unclear. This genomic interval coincides with a region with strong segregation distortion in some *A. arenosa* crosses, and it has been hypothesized to act as a female meiotic driver potentially harboring deleterious mutations (Dukić and Bomblies 2022). Alleles with transmission advantages are often less efficiently purged (Crow 1991; Taylor and Ingvarsson 2003; Lindholm *et al*. 2016; Zanders and Unckless 2019), which could explain why a potentially deleterious allele underlying the abnormal B772 phenotype has been retained in the Strecno population.

In the other families studied in detail, QTL analysis did not identify regions of the genome that were clearly linked to the abnormal phenotype. In these cases, multiple genomic regions might be involved, with different combinations of homozygous regions leading to the wide range of phenotypic abnormalities observed, and QTL analysis with more individuals might be required to identify the causal regions. This would be in agreement with observations of small-effect mutations distributed across multiple loci being maintained in natural populations at low frequencies, and collectively being a common source of inbreeding depression (Charlesworth and Willis 2009).

Perhaps the most intriguing case we analyzed is that of the B182 family, where a large fraction of individuals, more than a quarter, were phenotypically abnormal. One potential explanation could be that the family is fixed for one or more deleterious genes, but that the phenotype has only limited penetrance, i.e. is only revealed in a fraction of individuals, perhaps because of epigenetic or environmental instability. Other explanations are incompatible interactions between the nuclear and organellar genomes (Postel and Touzet 2020), or even vertical transmission of pathogens such as viroids (Venkataraman *et al*. 2021). While we lack RNA-seq data for this family, we attempted to test this hypothesis in the two families for which we have this data. In the A279 family, only a mild downregulation of genes associated with the GO term “response to virus” was observed (Supplementary Table 12), without such evidence in the B772 family.

## Conclusion

Our goal was to reveal the frequency and genetic basis of a set of deleterious phenotypes segregating in plants descended from open-pollinated wild *A. arenosa* individuals using a combination of transcriptome profiling, linkage mapping, and genome-wide ROH patterns. We show that deleterious phenotypes are present in around 10% of maternal sibships, and different genetic architectures likely underlie these deleterious phenotypes. In two out of four cases studied in detail, increased homozygosity in either one or several genomic regions is likely responsible for the observed phenotypic defects. These regions are prime candidates for harboring recessive deleterious alleles, although it remains to be shown how many such alleles are present in each of these regions, and how they interact. ROH identification is often exploited to identify recessive disease loci (Lencz *et al*. 2007; Sams and Boyko 2019) and for livestock breeding purposes (Peripolli *et al*. 2017; Dixit *et al*. 2020; Talebi *et al*. 2020). We highlight the use of ROH as a proxy for determining genomic regions likely contributing to inbreeding depression in wild plant populations. Our study complements recent ROH research conducted on cultivated pears and almonds (Kumar *et al*. 2020; Pavan *et al*. 2021). In conclusion, understanding how the combined action of specific alleles leads to deleterious phenotypes in wild populations, and how such genes and alleles are distributed, will be important for optimal use of genetic material in plant breeding.

## Data Availability

Sequencing data can be found at the European Nucleotide Archive (ENA) under project numbers PRJEB42608 (RNA-seq experiment, for phenotypes, see Supplementary Table 25), PRJEB42625 (*A. arenosa* assembly), GCA_905216605.1 (*A. arenosa* assembly and annotation), PRJEB67736 (RAD-seq of F_0_ individuals), PRJEB67742 (RAD-seq of F_2_ individuals for linkage mapping), PRJEB67945 (whole-genome sequencing of F_2_ B772 individuals), PRJEB67947 (whole-genome sequencing of F_2_ A279 individuals), PRJEB67951 (whole-genome sequencing of F_2_ B182 individuals) and PRJEB67954 (whole-genome sequencing of F_0_ individuals from Strecno). Phenotypes of F_0_ and F_2_ individuals sequenced by RAD-seq are in Supplementary Table 2. For phenotypes of whole-genome sequenced individuals see Supplementary Tables 5 (B772 F_2_), S21 (A279 F_2_), and S22 (B182 F_2_). For RNA-seq analysis of differential gene expression, raw read counts, processed read counts after normalization, and differential gene expression statistics can be found in Supplementary File 1 at https://doi.org/10.25387/g3.24581928.
